# Supplementary figures and images for: Nature-derived lignan compound VB-1 exerts hair growth-promoting effects by augmenting Wnt/β-catenin signaling in human dermal papilla cells
Source: PeerJ. 2018 May 8;6:e4737. doi: 10.7717/peerj.4737 (PMC5947041; doi:10.7717/peerj.4737)

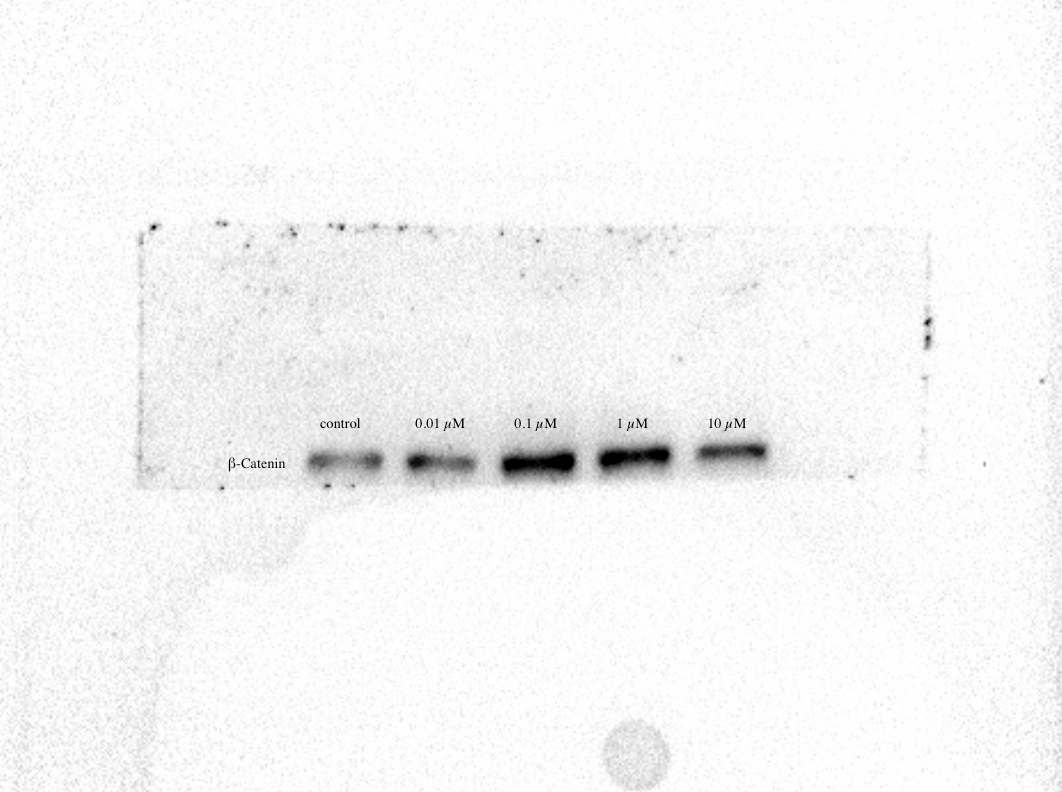

Supplement: Supplemental Information 1 [file peerj-06-4737-s001.png]

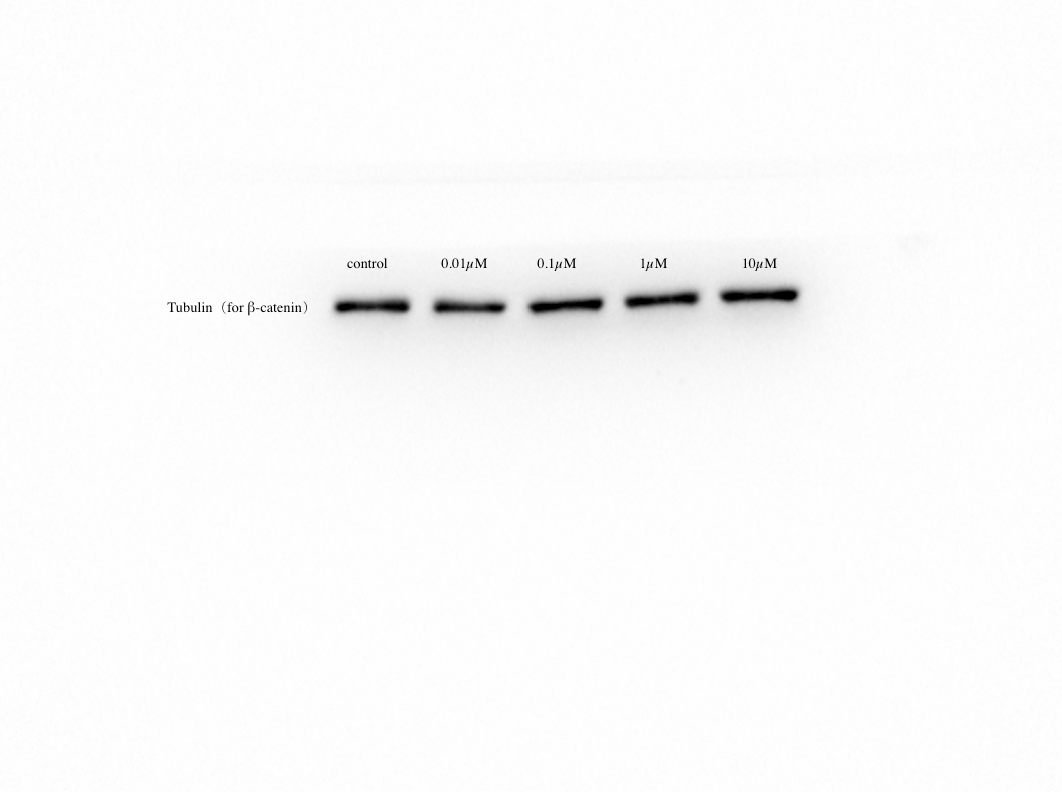

Supplement: Supplemental Information 2 [file peerj-06-4737-s002.png]

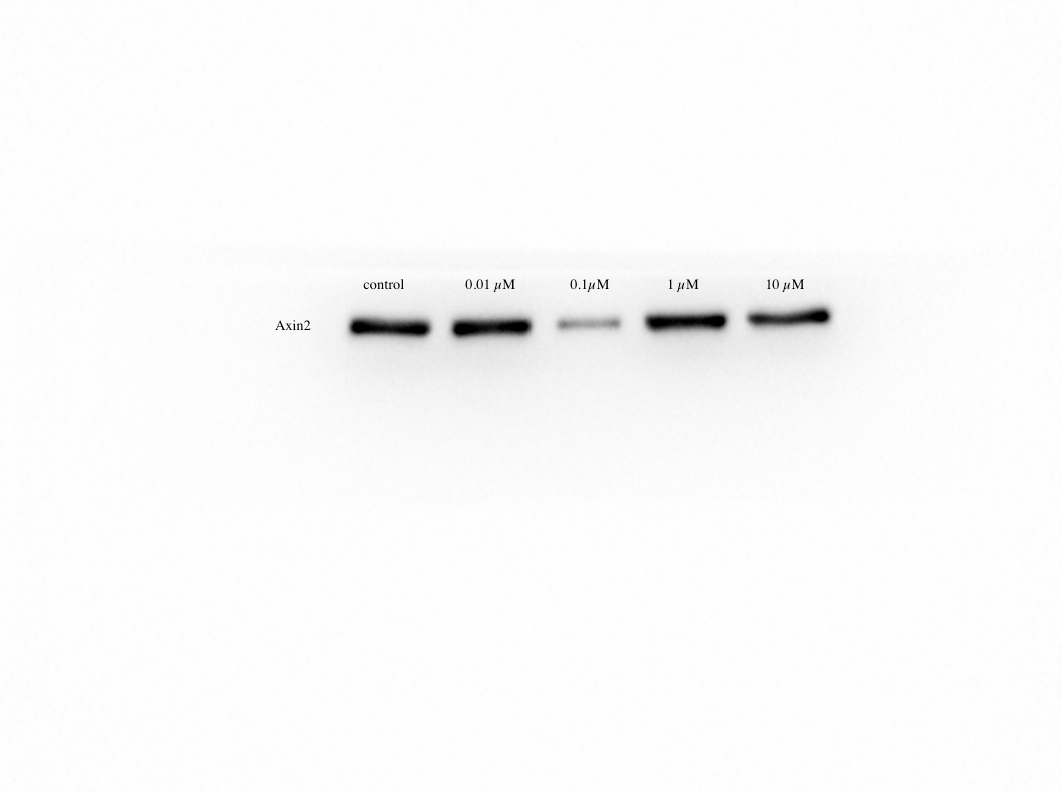

Supplement: Supplemental Information 3 [file peerj-06-4737-s003.png]

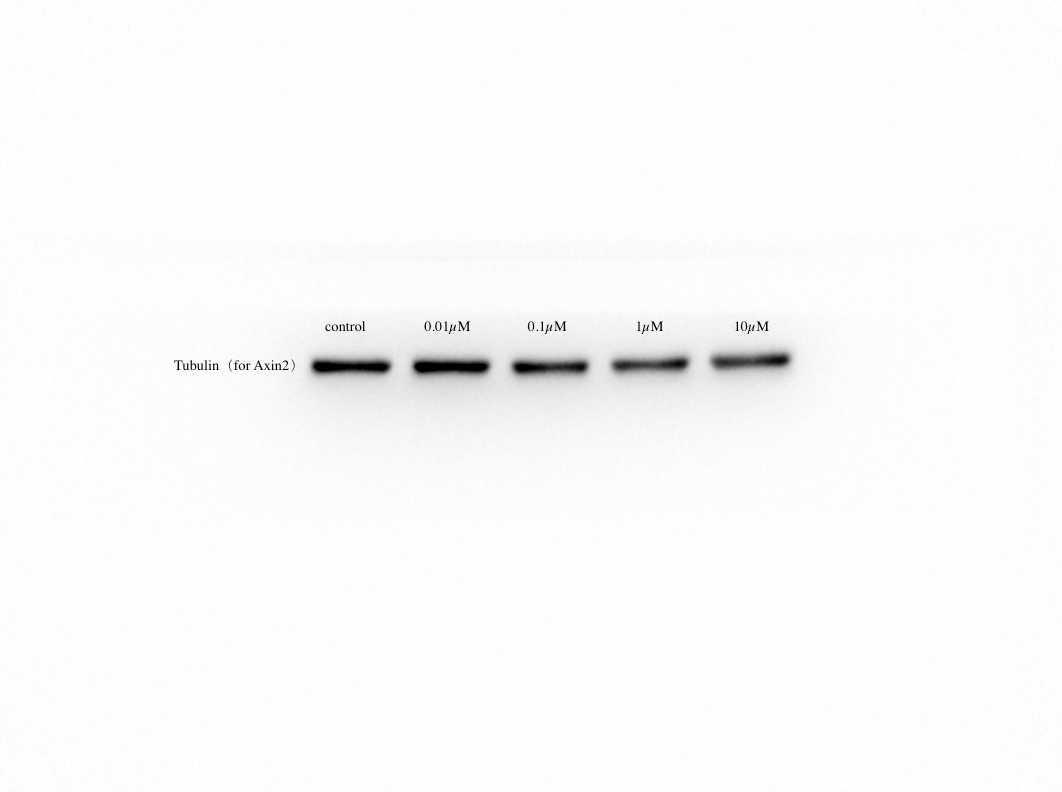

Supplement: Supplemental Information 4 [file peerj-06-4737-s004.png]

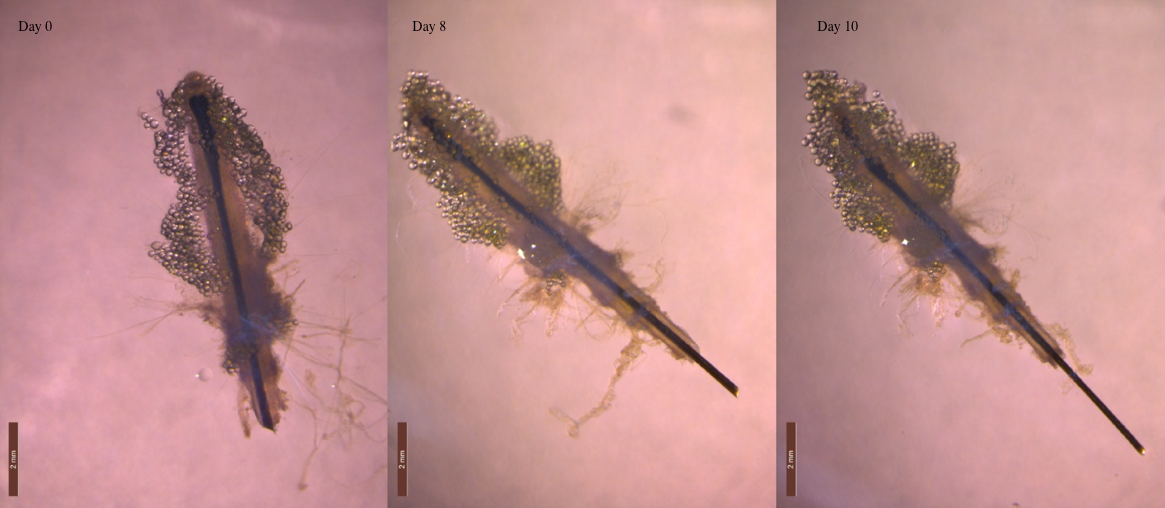

Supplement: Supplemental Information 5 [file peerj-06-4737-s005.png]
