# Supplementary material for: Nature-derived lignan compound VB-1 exerts hair growth-promoting effects by augmenting Wnt/β-catenin signaling in human dermal papilla cells
Source: PeerJ. 2018 May 8;6:e4737. doi: 10.7717/peerj.4737 (PMC5947041; doi:10.7717/peerj.4737)
Supplement: Table S1 — RNA and cDNA are extracted from cells using TRIzol reagent (Invitrogen, Carls- bad, CA) and RevertAid First Strand cDNA Synthesis Kit (Gibco/Thermo Scientific). Complementary DNA was synthesized from 1ng total RNA (Applied Bio-systems, CA, USA) using SYBR Green MasterMix (Applied-Biosystems-Thermofisher, Waltham, MA, USA). Sequences of qPCR primers were from PrimerBank followed by NCBI blast. [file peerj-06-4737-s006.docx]

| Target genes | Primer | Sequence |
| --- | --- | --- |
| Dkk-1 | forward | 5’-CCTTGAACTCGGTTCTCAATTCC-3’ |
|  | reverse | 5’-CAATGGTCTGGTACTTATTCCCG-3’ |
| Wnt5a | forward | 5’-TTGAAGCCAATTCTTGGTGGTCGC-3’ |
|  | reverse | 5’-TGGTCCTGATACAAGTGGCACAGT-3’ |
| Lef1 | forward | 5’-GAATTAGCACGGAAAGAAAGA-3’ |
|  | reverse | 5’-ACCTGTACCTGATGCAGATT-3’ |
| Axin2 | forward | 5’-CAAACTTTCGCCAACCGTGGTTG-3’ |
|  | reverse | 5’-GGTGCAAAGACATAGCCAGAACC-3’ |
| BMP2 | forward | 5’-AGCAACGCTAGAAGACAGC-3’ |
|  | reverse | 5’-TGCTTCTTAGACGGACTGCG-3’ |
| BMP4 | forward | 5’-TAGCAAGAGTGCCGTCATTCC-3’ |
|  | reverse | 5’-GCGCTCAGGATACTCAAGACC-3’ |
| GAPDH | forward | 5’-TGTTGCCATCAATGACCCCTT-3’ |
|  | reverse | 5’-CTCCACGACGTACTCAGCG-3’ |
